# Self-Transcendent Wisdom Mediates the Association Between Spirituality and Well-Being in Six Nations

**DOI:** 10.1093/geroni/igab046.507

**Published:** 2021-12-17

**Authors:** Monika Ardelt, Juensung Kim, Michel Ferrari

**Affiliations:** 1 University of Florida, Gainesville, Florida, United States; 2 University of Toronto, Toronto, Ontario, Canada

## Abstract

Distraught individuals sometimes turn to religion for solace, particularly in old age, so spirituality is not necessarily positively related to well-being. However, spirituality might lead to greater well-being if it promotes self-transcendent wisdom. Using a sample of 307 respondents from six nations (USA, Canada, Serbia, Ukraine, Iran, and China), ranging in age from 59 to 99 years (M=73.00, SD=8.13), this study tested the generalizability of the hypothesized mediated pathway. Results showed only weak correlations between spirituality and well-being measures in the whole sample. Yet, as predicted, spirituality, mediated by self-transcendent wisdom, was indirectly related to greater well-being in all six nations, despite significant differences by nation in variable means. Spirituality had additional direct positive effects on life satisfaction in Canada, Iran, and China and on general well-being in Iran and China. These findings suggest that spirituality likely results in greater well-being when it transcends egocentric concerns.

